# Comparison of decision tree and naïve Bayes algorithms in detecting trace residue of gasoline based on gas chromatography–mass spectrometry data

**DOI:** 10.1093/fsr/owad031

**Published:** 2023-09-19

**Authors:** Md Gezani Bin Md Ghazi, Loong Chuen Lee, Aznor S Samsudin, Hukil Sino

**Affiliations:** Forensic Science Program, CODTIS, Faculty of Health Science, Universiti Kebangsaan Malaysia, Selangor, Malaysia; Fire Investigation Division, Fire and Rescue Department of Malaysia, Putrajaya, Malaysia; Forensic Science Program, CODTIS, Faculty of Health Science, Universiti Kebangsaan Malaysia, Selangor, Malaysia; Institute of IR 4.0, Universiti Kebangsaan Malaysia, Selangor, Malaysia; Fire Investigation Laboratory, Fire Investigation Division, Fire and Rescue Department of Selangor, Selangor, Malaysia; Forensic Science Program, CODTIS, Faculty of Health Science, Universiti Kebangsaan Malaysia, Selangor, Malaysia

**Keywords:** forensic sciences, gas chromatography–mass spectrometry, forensic fire debris analysis, naïve Bayes classifier, classification and regression tree, principal component analysis

## Abstract

Fire debris analysis aims to detect and identify any ignitable liquid residues in burnt residues collected at a fire scene. Typically, the burnt residues are analysed using gas chromatography–mass spectrometry (GC–MS) and are manually interpreted. The interpretation process can be laborious due to the complexity and high dimensionality of the GC–MS data. Therefore, this study aims to compare the potential of classification and regression tree (CART) and naïve Bayes (NB) algorithms in analysing the pixel-level GC–MS data of fire debris. The data comprise 14 positive (i.e. fire debris with traces of gasoline) and 24 negative (i.e. fire debris without traces of gasoline) samples. The differences between the positive and negative samples were first inspected based on the mean chromatograms and scores plots of the principal component analysis technique. Then, CART and NB algorithms were independently applied to the GC–MS data. Stratified random resampling was applied to prepare three sets of 200 pairs of training and testing samples (i.e. split ratio of 7:3, 8:2, and 9:1) for estimating the prediction accuracies. Although both the positive and negative samples were hardly differentiated based on the mean chromatograms and scores plots of principal component analysis, the respective NB and CART predictive models produced satisfactory performances with the normalized GC–MS data, i.e. majority achieved prediction accuracy >70%. NB consistently outperformed CART based on the prediction accuracies of testing samples and the corresponding risk of overfitting except when evaluated using only 10% of samples. The accuracy of CART was found to be inversely proportional to the number of testing samples; meanwhile, NB demonstrated rather consistent performances across the three split ratios. In conclusion, NB seems to be much better than CART based on the robustness against the number of testing samples and the consistent lower risk of overfitting.

## Introduction

Forensic fire investigation consists of determining the origin, ignition source, cause, and development of fire [1]. In general, the success in fire investigation depends on both field investigation and laboratory-based analysis to determine and identify ignitable liquids (ILs) found at the scene [2]. ILs, including gasoline, kerosene, and diesel, are among the few frequently encountered in a fire scene, with gasoline emerging to be the most popular one attributed to its combustion efficiency, readiness, and low cost [3–5]. ILs are essentially a mixture of volatile organic compounds (VOCs) and thus can be detected using gas chromatography–mass spectrometry (GC–MS). The GC–MS data of ILs are laborious to be interpreted caused by its inherent complex composition and matrix effects, e.g. thermal decomposition or pyrolysis of the matrix [6–10].

In practice, American Society for Testing and Materials (ASTM) Standard E1618 [11] has established a number of markers for identifying gasoline, diesel, and kerosene based on GC–MS data. The guideline describes interpretation procedures involving the extracted ion chromatogram (EIC) and total ion chromatogram (TIC) of the GC–MS output. EIC presents the total ion counts of a particular or predefined range of *m/z* value over the retention time. Meanwhile, TIC shows the total ion counts over the full range of studied *m/z* value [12]. Mathematically, EIC is the subset of TIC.

More recently, chemometric techniques have been widely studied in fire debris analysis to aid analysts in interpreting the complicated TICs of burnt residues [13, 14]. For instance, Sinkov et al. [4] reported the use of partial least squares-discriminant analysis (PLS-DA) and soft independent modelling of class analogies methods in discriminating samples containing and without gasoline. Recently, Allen et al. [15] integrated likelihood ratios into a PLS-DA model for predicting the presence of ignitable liquid residues (ILRs) based on TICs collected from 9 000 simulated fire debris. The authors reported that the PLS-DA model could be a feasible model for the forensic analysis of fire debris data. On the other hand, Ugena et al. [16] evaluated the potential of neural networks in discriminating fuels based on gas chromatography-flame ionization detector data.

To date, and to the best of our knowledge, there is no such research comparing the classification and regression tree (CART) and naïve Bayes (NB) algorithms using pixel-level GC–MS data, particularly in the context of fire debris analysis. Hence, this work aims to compare the performance of CART and NB in predicting the presence of gasoline in fire debris based on the pixel-level GC–MS data. In addition, principal component analysis (PCA) was also performed to explore the data prior to the predictive modelling. Due to the small sample size, model performances including prediction accuracy and its variability, and overfitting of model have been derived using the stratified random resampling method.

## Materials and methods

The GC–MS data of fire debris were obtained from the Fire Investigation Laboratory of Selangor, Fire and Rescue Department of Selangor (FRDS), Shah Alam, Malaysia with permission for using in this study. All the fire debris was collected for fire cases happening from November until December 2020 in Selangor, Malaysia. The GC–MS profiles of the fire debris have been prepared by a certified chemist based at FRDS, Malaysia. Both the sample preparation and GC–MS methods are adopted from the ASTM standards, i.e. E1618 [11] and E2154-15a [17]. The following sections describe the analytical procedures in detail.

### Burnt residue sampling

The collection of real fire debris samples has been performed by the fire investigator of the FRDS, Malaysia. All the fire debris were separately kept in nylon arson evidence bags (Tri-tech Forensics, Executive Park Blvd, Southport, NC, USA) and fastened tightly to avoid VOCs loss. Nylon bags are commonly used for fire debris collection since they are easy for storage and transportation [2].

### Headspace concentration-solid phase microextraction and GC–MS analysis

The ILRs of the samples were first extracted using a headspace concentration-solid phase microextraction (HS-SPME) procedure. A 65 μm polydimethylsiloxane-divinylbenzene (PDMS/DVB) fibre housed in a manual SPME holder (Supelco, Bellefonte, PA, USA) was used for the extraction. To ensure no component peak arose from the SPME membrane, the fibre blank was first introduced into the GC–MS system and analysed according to the method used for analysing ILRs extracted from the fire debris samples. Given a fire debris sample, headspace was first created by keeping it in an oven at 90°C for 15 min. Then, the nylon bag was removed from the oven and subsequently pierced with the SPME needle. After 20 min, the SPME needle was removed and directly inserted into the gas chromatographic injector port. An SPME fibre blank was performed each time before a new sample injection to ensure no carryover of the previous sample.

All the GC–MS analyses were performed on a PerkinElmer Clarus® 680 gas chromatography coupled with a Clarus® SQ 8T mass spectrometry (Waltham, MA, USA). Table 1 presents the parameters adopted to perform the GC–MS analysis. The GC–MS data were acquired by a PerkinElmer TurboMass (version 6.1.0).

**Table 1:** Parameters of gas chromatography-mass spectrometry method.

Parameter	Description
Column	Elite-5MS (30 m × 250 μm (I.D.), 0.25 μm)
Carrier gas	Helium, 1.0 mL/min
Inlet temperature	200°C
Initial temperature	50°C
Initial hold time	2.5 min
Ramp rate	15.0°C/min
Final temperature	300°C
Final hold time	5.82 min
Mass spectrum detection transfer line temperature	200°C
Source temperature	200°C
Scan range	45–450 amu

### Interpretation of fire debris

All the fire debris samples were examined for any ILRs by a certified chemist based at FRDS. Given a sample, the overall TIC pattern is first visually inspected to look for any prominent markers of IL by comparing it against a TIC of neat IL, e.g. gasoline (prepared on the same day as the sample using the same methods and GC–MS system). For a sample showing a sufficient number of prominent IL markers, further examination will involve chemical identification of the markers *via* mass spectral library searching. In case the TIC of the sample is heavily masked by peaks from pyrolysis product or background, EIC of selected compounds, as stated in ASTM standard E1618 [11], is prepared for both samples and neat IL, then, they are compared for the presence of important markers of the IL. Sample detected with gasoline is labelled as ``ILR detected'' or otherwise as ``ILR not detected''. IL detected in a fire debris is then further classified into one of the types defined by ASTM standard E1618, i.e. gasoline, petroleum distillates (kerosene, diesel fuel, cigarette lighter fluids), isoparaffinic products (aviation gas), aromatic products (toluene-based products), naphthenic-paraffinic products (solvent), normal alkane products (candle oils, lamp oil), mixture oxygenated solvents (alcohols, ketones), and others–miscellaneous (turpentine products).

### Preparing TIC data

For the sake of brevity, all negative samples and only fire debris detected containing gasoline were studied herein. This study replaced the initial labels, so fire debris detected with gasoline was known as positive and otherwise as negative samples. The raw format of all selected samples was first exported from the desktop connected to the GC–MS system and saved in an external hard disc. Next, the data were imported into another working desktop and converted into a format that can be processed by a statistical software. First, the .raw data were converted into mzXML format *via* MSconvert software [18, 19]. Next, the resulting mzXML data were imported into Mass^++^ software [20] to obtain the full TIC data in .txt format. All the .txt files were arranged in the form of a matrix and saved in a Microsoft Excel® (Denver, CO, USA) spreadsheet.

The final data matrix is composed of 38 chromatograms, i.e. TICs, and readily be classified into two classes: (i) 14 positive (gasoline detected); and (ii) 24 negative (no gasoline detected). Each chromatogram was presented with 4 999 variables. The sampling point of the GC–MS was set in such a way that each chromatogram covering retention time from 0 to 25 min was eventually presented with 4 999 intensity values.

### Statistical data analysis

All the statistical analyses were accomplished in the R statistical software, v. 3.6.2 [21]. First, the mean chromatograms of positive and negative samples were computed using an in-house function. Then, PCA [22, 23] was performed using the function prcomp to explore the data matrix *via* scores plots. Meanwhile, CART and NB modelling were executed using codes provided by e1071 [24] and rpart [25] packages, respectively.

### Normalization

In practice, the SPME method does not allow the researcher to determine the quantity of extract injected into the GC–MS system. Hence, it is important to first eliminate bias due to the unequal initial amount of sample *via* normalization by converting the signals into relative values [26, 27]. Normalization to sum was performed by dividing each of the 38 TICs with the respective sum of 4 999 intensity values (of 4 999 variables).

### CART

Several recently published articles have demonstrated the success of the decision tree model in solving forensic problems, e.g. sex estimation [28, 29] and paper discrimination [30]. However, there has been no attempt to evaluate the potential of decision tree in modelling GC–MS data of fire debris. CART is one of the most used algorithms to construct a decision tree model [31]. The algorithm aims to split the data recursively until reaching purity. Herein, the CART algorithm splits the TIC data into either positive or negative classes according to a specific variable (i.e. retention time point). The splitting is governed by the Gini coefficient that evaluates the reduction of heterogeneity [32].

### NB

NB classifier is far more popular than the CART and was also proven to be useful in the field of forensic science, e.g. forensic anthropology [33]. Recently, Bogdal et al. [34] demonstrated the merits of various machine learning algorithms to detect gasoline in fire debris. Even though NB has been evaluated in their work, the relative performance between NB and CART in modelling GC–MS data of fire debris was not of their concern. Thus, this is the first work comparing NB with CART in fire debris analysis. NB classification relies on the Bayes rule [35] as expressed by Equation (1):


(1) \begin{equation*} P\!\left(y|x\right)=\frac{P\!\left(x|y\right)P(y)}{P(x)} \end{equation*}

where *x* denotes a particular retention time; *y* refers to the presence of gasoline in the sample, i.e. positive or negative. In this work, the ratio of positive and negative samples was unbalanced. Thus, the class *a priori* probabilities, *P*(*x*), played a role in the modelling problem. The $P\!\left(x|y\right)$ was determined according to the training data. The NB model was used in predicting the presence of gasoline in a fire debris sample (*y*) submitted to the laboratory. Given a TIC of an unknown burnt residue, the presence of gasoline (*y*) is predicted as positive if the posterior probability of the positive class $P\!\left({y}_{positive}|x\right)$ is higher than the negative class $P\!\left({y}_{negative}|x\right)$.

### Model validation

To minimize bias caused by the small sample size and unbalanced group sizes, stratified random resampling was adopted to prepare varying pairs of training and test sets. The random sampling was separately performed in positive and negative samples, i.e. stratified random resampling, to ensure both the training and testing samples have the same proportion of classes. The resampling was performed at three split ratios (i.e. 7:3, 8:2, and 9:1), respectively, repeated 200 times. Then, the prediction accuracies were estimated using the training (Acc_AP_) and testing (Acc_ET_) samples, Equations (2) and (3):


(2) \begin{equation*} {\mathrm{Acc}}_{\mathrm{AP}}=\frac{n_{\mathrm{train}}^{\ast }}{n_{\mathrm{train}}}\kern1.25em \left({n}_{\mathrm{train}}^{\ast}\le n\right) \end{equation*}

(3) \begin{equation*} \kern-.5pc{\mathrm{Acc}}_{\mathrm{ET}}=\frac{n_{\mathrm{test}}^{\ast }}{n_{\mathrm{test}}}\kern1.75em \left({n}_{\mathrm{test}}^{\ast}\le n\right) \end{equation*}

where *n* and n* refer to the total of (training/testing) samples and number of samples correctly predicted by the model.

Eventually, the NB and CART models were presented with three series of prediction accuracy by the training and testing samples. Hence, the predictive capability of a model was evaluated based on the average of the 200 prediction accuracies by the three split ratios, see Equation (4). Meanwhile, the variability of the 200 prediction accuracies was assessed graphically by beeswarm plot. Squared of difference between prediction accuracies of training and testing samples (SDP) denoted by Equation (5) was determined for evaluating the risk of overfitting [36].


(4) \begin{equation*}\kern-3.5pc \overline{\mathrm{Acc}}=\frac{1}{R}\left(\sum \limits_{r=1}^R{\mathrm{Acc}}_r\right) \end{equation*}

(5) \begin{equation*} \mathrm{SDP}=\frac{1}{R}\sum \limits_{r=1}^R{\left( {\rm Ac{c}_{AP}}- {\rm Ac{c}_{ET}}\right)}^2 \end{equation*}

## Results

### Mean chromatogram

**Figure 1:**
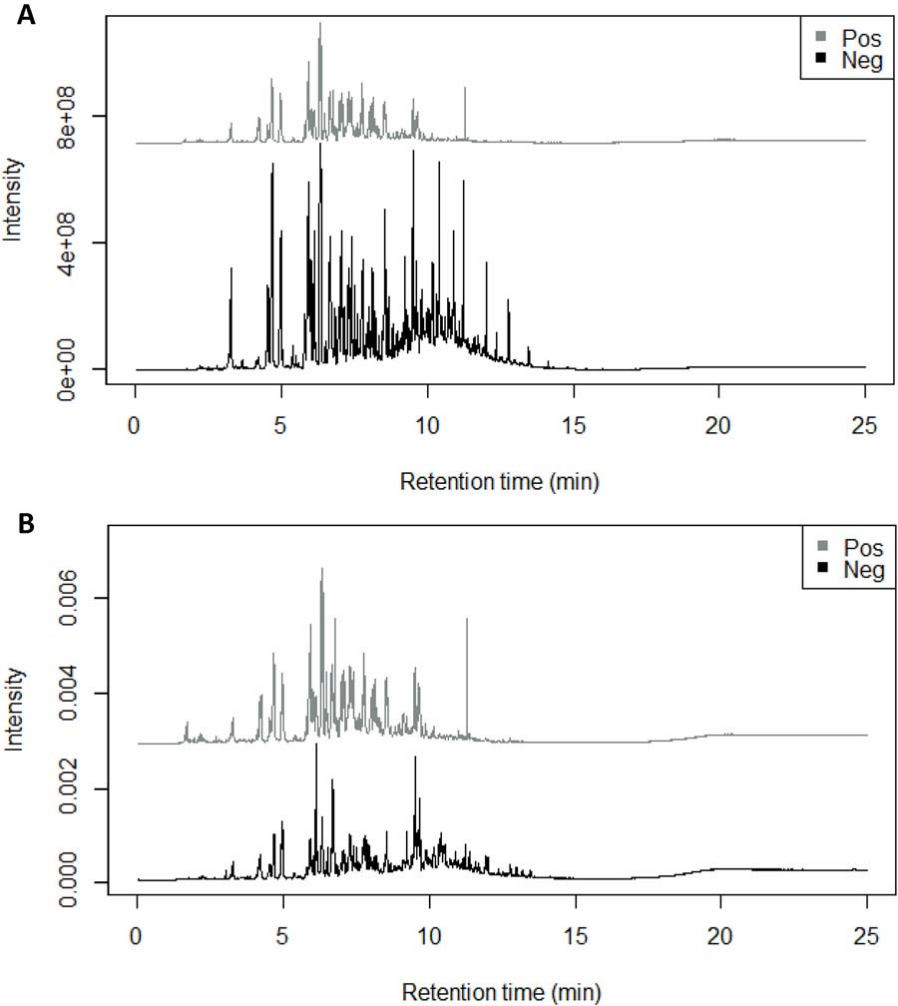
Mean chromatograms of the (A) raw and (B) normalized total ion chromatogram. Pos: positive; Neg: negative.

Generally, it is most desired that the positive and negative samples showed highly different chromatographic profiles. Figure 1A and B illustrates the raw and the corresponding normalized mean chromatograms of the positive and negative samples, where each was averaged from 14 to 24 samples, respectively. Basically, the negative samples were found to show slightly more peaks than the positive ones. The peaks were contributed by volatile compounds extracted from fire debris but did not originate from the ILs. According to Sandercock [37], pyrolysis, combustion, and distillation typically happened in a fire scene. These processes often produce varying numbers of volatile and semi-volatile background compounds similar to VOCs of ILs.

Meanwhile, both the positive and negative samples presented a high number of peaks eluting between 0 and 15 min. It is worth noting that the peak heights were comparable between positive and negative samples after the data were normalized (Figure 1B). Prior to normalization, the peak height was found to be dependent on the initial quantity of the SPME extract. Though, the overall peak distributional patterns observed remained unchanged. This is expected since normalization only converts the signals from absolute values to relative ones. In the following sections, only results obtained from the normalized data were presented since the raw data were found to be flawed herein.

### Scores plot of PCA

**Figure 2:**
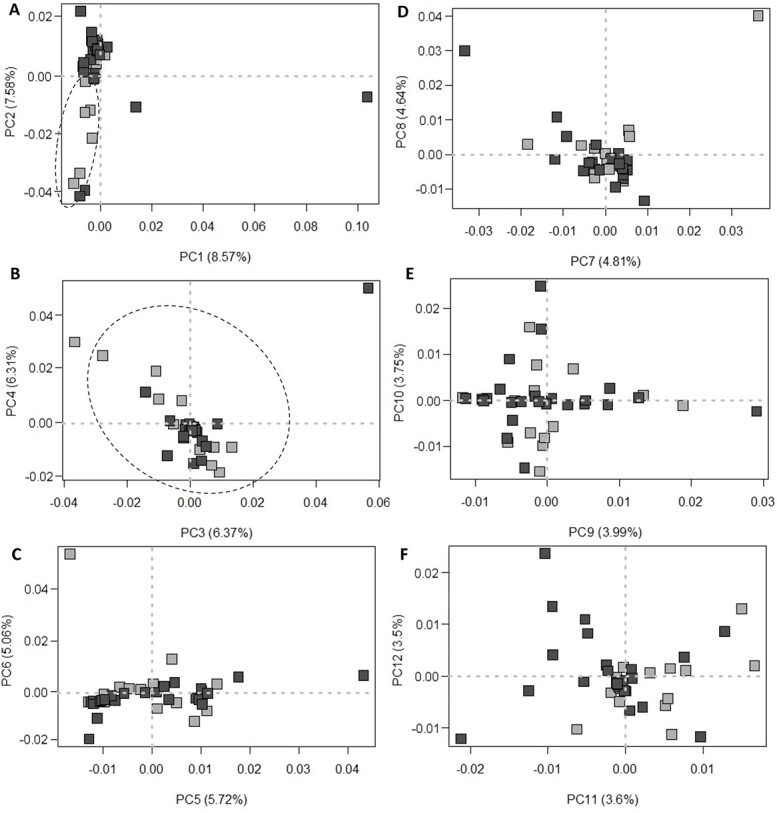
Scores plots of principal component analysis (PCA) computed using the normalized total ion chromatogram (TICs) constructed using the first 12 PCs: (A) PC1 *vs* PC2, (B) PC3 *vs* PC4, (C) PC5 *vs* PC6, (D) PC7 *vs* PC8, (E) PC9 *vs* PC10, and (F) PC11 *vs* PC12 light gray square: positive samples; dark gray square: negative samples.

Next, the spatial distribution of the negative and positive samples after normalization was inspected using scores plots of PCA. The normalized TIC data were mean centred before processing by PCA. Mathematically, mean-centring does not change the nature of the data but ease the interpretability of the results [38]. Figure 2 shows the six 2D score plots of PCA computed from the first 12 PCs. Although none of the PCs showed a perfect separation between the positive and negative samples, the second PC (PC2) split most samples into the two groups. The remaining PCs have the samples scattered widely without any clear grouping by the presence of gasoline.

Since PCA identifies the linear combination (of 4 999 variables) i.e. called the PC with maximum variance in the dataset, it is always expected the most desired separation would be obtained with PC1 and then PC2. However, PC2 was found to show a better separation between the positive and negative samples than that obtained using PC1. Thus, it seems sound to postulate here that only the minority variance of the TIC data helps explain the difference between the positive and negative samples. Most of the variance is irrelevant in explaining the difference between the positive and negative samples.

### Performances of predictive models

**Figure 3:**
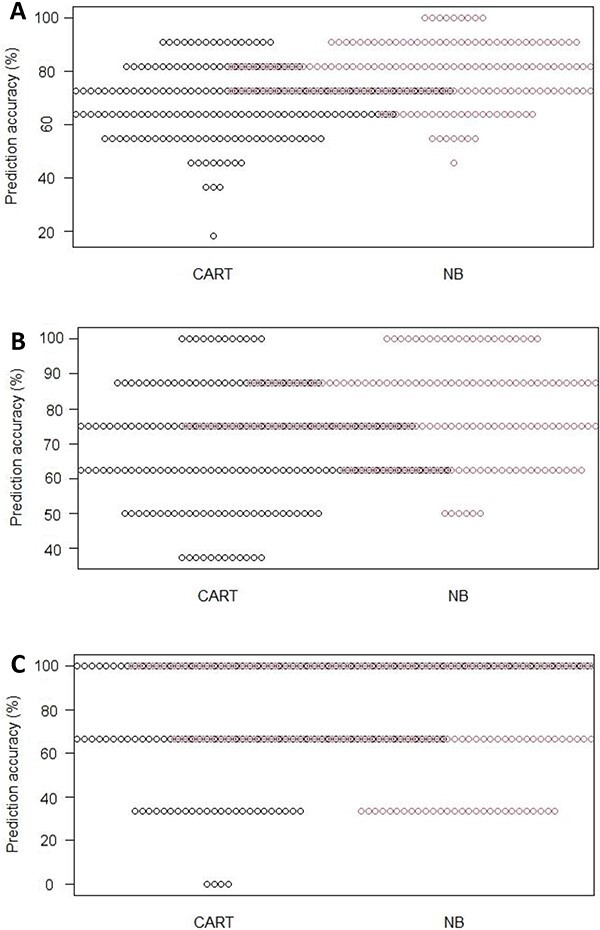
Beeswarm plots showing variability of 200 prediction accuracies estimated using the testing samples presented by classification and regression tree (CART) and naive Bayes (NB) models and split ratio: (A) 7:3, (B) 8:2, and (C) 9:1.

Table 2 shows the individual class and overall mean prediction accuracies of testing samples as well as the SDP values by the split ratio (i.e. 7:3, 8:2, and 9:1) and modelling algorithms (CART and NB). A lower SDP value is preferred herein since it indicates the model would perform well in a real-world setting [36]. Meanwhile, Figure 3 illustrates the variability of the 200 prediction accuracies estimated from the testing samples between the CART and NB models using beeswarm plots and presented by the three split ratios.

**Table 2:** Average of 200 prediction accuracies of testing samples and the respective SDP value.

Split ratio	Model	Prediction accuracy			SDP
		Negative	Positive	Overall	
7:3	CART	73.07	60.00	68.32	0.0246
	NB	79.29^a^	76.38^a^	78.23^a^	0.0217^a^
8:2	CART	73.20	60.83	68.56	0.0246
	NB	79.60^a^	77.00^a^	78.63^a^	0.0217^a^
9:1	CART	76.75^a^	84.50^a^	79.33^a^	0.0238
	NB	76.50	79.00	77.33	0.0238

a The more outstanding model. SDP: squared of difference between prediction accuracies; CART: Classification and regression tree; NB: naïve Bayes.

Despite the fact that none of the models has achieved 100% prediction accuracy, the majority of the models achieved accuracy >70%, denoting the potential of machine learning in predicting the presence of gasoline. It is noted that the prediction accuracy of CART was inversely proportional to the number of testing samples, i.e. the highest accuracy was achieved using 10% of samples (79.33%). The outperformance of CART derived using only 10% of samples is attributed to its better capability in predicting positive samples (Figure 3C). On the other hand, the performance of NB was relatively consistent across the three different split ratios, with the highest accuracy derived using 20% of samples (78.63%).

Overall, NB seems to be outperformed CART based on three grounds. First, NB achieved higher accuracy in two of the three split ratios. Although the highest accuracy was obtained by CART, the challenge of predicting 10% of the sample is definitely lower than predicting 20% or 30% of the samples. Next, CART achieved only an additional 2% accuracy rate than the NB (in the 9:1 set); meanwhile, NB always showed an additional 9% accuracy than the CART (in 7:3 and 8:2 sets). Last but not least, NB demonstrated better potential than CART in terms of risk of overfitting; NB showed the same risk of overfitting as CART even though the latter achieved higher prediction accuracy. On the contrary, CART had always presented a higher risk of overfitting when NB achieved higher prediction accuracy.

## Discussions

This work is the first report on relative performances of CART and NB algorithms in modelling TICs for detecting ILRs. Even though the data comprised only 38 samples, the predictive models have been intensively evaluated *via* stratified random resampling method. Both the algorithms were compared according to three aspects: (i) prediction accuracy, (ii) variability of the prediction accuracy, and (iii) risk of overfitting.

Generally, NB was tended to outperform the CART algorithm in modelling the binary problem of TIC data. As mentioned above, NB determines the class of an unknown sample based on posterior probability computed from all the input variables and incorporating an *a priori* probability [39]. Meanwhile, CART is an ensemble method composing variable selection and predictive modelling [40]. Hence, the outperformance of NB demonstrated herein can be explained by two rationales.

Attributed to the higher ratio of sample in the negative group, both CART and NB models tended to obtain higher prediction accuracy with the negative class, except when tested using only 10% of the samples. However, the relative difference between positive and negative classes’ accuracies was more prominent in the CART models than in the NB models. NB models are believed to have minimized the discrepancy by incorporating the unequal *a priori* into the posterior probability. On the other hand, the CART algorithm cannot minimize the bias in predicting the testing samples.

Theoretically, CART has not considered all the 4 999 variables concurrently in predictive modelling but selects a few variables to construct the model. On the contrary, NB computes the posterior probability of a given class from all the 4 999 variables. By referring to Figure 1, one can see that most of the peaks were not sharp or fully resolved as a single peak, i.e. a single retention time point was insufficient to denote a peak. Since CART considers only a retention time point at a time, this explains the underperformance of the CART algorithm compared with the NB algorithm in this work.

Even though the NB models were found to outperform CART models, it is worth mentioning that the external prediction accuracy of NB models never reached >80%. This could be partially explained by the fact that the TIC data did not comply with at least two assumptions of the NB algorithm, i.e. normal distribution and independent variables. In this study, the variables referred to the 4 999 intensity values of each chromatogram. As highlighted above, none of the peaks is truly well resolved and resembles a sharp peak in the TIC; thus, it is sensible to assume adjacent intensity values contributing to a peak are dependent on each other. Next, the normality assumption can hardly be fulfilled due to the relatively small sample size.

## Limitation

There is an informal consensus that chromatographic data are inherently affected by drifted retention time. As such, peak alignment is often performed to minimize variations caused by the limitation. Despite a wide range of peak alignment algorithms proposed in the literature, most of them work on a target or reference chromatogram, e.g. chromatograms of a standard sample or a mean chromatogram of replicate samples [41]. As described above, the data were provided by the FDRM without a chromatogram of standard sample, and each sample was analysed only once without any replicate available to us. Consequently, the data have not been corrected using any peak alignment algorithm due to the technical constraint. Though the absolute performance values of CART and NB reported herein could have been improved after peak alignment, their relative performances presented above are still valid.

## Conclusions

In conclusion, NB is much better than CART based on the robustness against the number of testing samples and the consistent lower risk of overfitting. Despite the fact that peak alignment was not performed, and the data were also limited in sample size, the relative performances of the two modelling algorithms reported herein are reliable since a stratified random resampling method has been deployed to derive the model performances. In future work, the true predictive capability of the NB algorithm in pixel-level GC–MS data of fire debris shall be estimated using a bigger sample size, and peak alignment must be executed before predictive modelling.
